# The Management of Dental Practices in the Post-COVID 19 Era: An Economic and Operational Perspective

**DOI:** 10.3390/ijerph17238905

**Published:** 2020-11-30

**Authors:** Giovanna Lo Nigro, Maria Eleonora Bizzoca, Lorenzo Lo Muzio, Giuseppina Campisi

**Affiliations:** 1 Department of Engineering, University of Palermo, 90128 Palermo, Italy; giovanna.lonigro@unipa.it; 2 Department of Clinical and Experimental Medicine, University of Foggia, 71122 Foggia, Italy; marielebizzoca@gmail.com; 3 C.I.N.B.O. (Consorzio Interuniversitario Nazionale per la Bio-Oncologia), 66100 Chieti, Italy; 4 Department of Surgical, Oncological and Oral Sciences (DiChirOnS), University of Palermo, 90127 Palermo, Italy; campisi@odonto.unipa.it or

**Keywords:** management, economics, COVID-19, dentistry, SARS-CoV-2

## Abstract

Background: In order to protect dental teams and their patients during the COVID-19 pandemic, dentists have had to adopt several measures (operating and post-operating procedures) which may increase the total treatment time and costs relating to individual protective measures. This paper will propose a thorough analysis of operating dentistry procedures, comparing the economic performance of the activity in a dental surgery before and after the adoption of these protective measures, which are required to contain the risk of SARS-COV-2 infections. Methods: The economic analysis is articulated in three approaches. Firstly, it assesses a reduction in markup by maintaining current charges (A); alternatively, it suggests revised charges to adopt in order to maintain unvaried levels of markup (B). And the third Approach (C) examines available dental treatments, highlighting how to profitably combine treatment volumes to reduce markup loss or a restricted increase in dental charges. Results: Maintaining dental charges could cause a loss in markup, even rising to 200% (A); attempting to maintain unvaried levels of markup will result in an increase in dental charges, even at 100% (B); and varying the volumes of the single dental treatments on offer (increasing those which current research indicates as the most profitable) could mitigate the economic impact of the measures to prevent the transmission of SARS-COV-2 (C). Conclusions: The authors of this paper provide managerial insights which can assist the dentist-entrepreneur to become aware of the boundaries of the economic consequences of governmental measures in containing the virus infection.

## 1. Introduction

From the first days of the containing of the COVID-19 pandemic, dental activity throughout the world has been drastically interrupted and thereafter characterised by uncertainty and subject to revised measures. The latter were included in recommendations provided by single governing bodies [1,2,3]. In order to obviate such challenges, the dentist has been obliged to adopt a series of protective measures, all of which are having a marked financial impact on finances and a lengthening of treatment times. This is the sum of time required for operating, and pre- and post-operating procedures, currently leading to generic economic loss, and leading to much uncertainty regarding future of the profession. In brief, maintaining the maximum level of SARS-CoV-2 safety would lead to a marked increase in costs and a reduction in the maximum number of treatments, which can be offered to patients on the timescale of reference (usually one year).

As discussed in a recent McKinsey report [4], business leaders should initially determine the scale, pace, and degree of intervention required in addressing one of the most far-reaching humanitarian crises of our time. This report can be considered as a contribution to this phase in assisting dentists to comprehend the extent of the effects of the COVID crisis. 

Small businesses are the most threatened by the economic crisis created by COVID-19. An American survey by the Becker Friedman Institute [5] has demonstrated how many businesses can be considered to be financially vulnerable: “The median firm with expenses over $10,000 per month has only enough cash on hand to last for two weeks. Three-quarters of respondents state that they only have enough cash on hand to cover two months of expenses or less”. 

The aim of this research is to assist the dentist-owner of a dental practice in their attempt to overcome the financial crunch, which may arise as a consequence of the adoption of anti-COVID measures.

The authors of this paper have made an analysis of the production and economic performance of a dentist’s practice in order to assess the economic impact of the aforementioned recommendations in limiting the transmission of SARS-CoV-2. Furthermore, the result of such an analysis places the dentists at the centre of a systematic analysis of their activity regarding operating/commercial choices. It will no doubt be of interest to the dentist as an entrepreneur to foresee the degree of predicted losses if pre-COVID charges are adhered to or how much to increase dental charges in maintaining markup levels. These tasks will be accomplished by adopting Approaches A and B, as proposed in this paper. In brief, Approach A assesses the loss in markup leaving the charges for dental treatments unchanged, while Approach B investigates the reverse situation: that is, maintaining unvaried the markup and assessing the increase in the charges for dental treatments. Having delimited the range of economic performance in terms of markup and dental charges with the first two approaches, it is of interest to address another variable available to the dentist entrepreneur; namely, the typologies and availability of related volumes of treatments. With the third approach, C, the authors will demonstrate that economic results depend on treatment volumes. Indeed, this third approach will demonstrate how to modify the dental treatment mix available (product mix) so as to contain the loss in economic performance. The authors would like to suggest how to combine these treatments by favouring those with a lesser differential economic impact (i.e., those which impact less on pre/post-COVID in terms of turnover or profit) or the more profitable treatments under the new scenario arising from the adoption of anti-COVID recommendations. The authors of this paper hope that it may be considered as pioneering in the body of literature, which can only flourish. Indeed, a rapid reaction can be the keystone of the survival of small businesses when times are harsh. To the best of the authors’ knowledge, only Schwendicke et al [6] have hitherto researched this field to provide economic insights into the post-COVID scenario of a dental surgery. Their paper analyses the German healthcare service from the perspective of a dental provider. While Schwendicke et al. [6] have focused on the differential economic impact of Covid-19 as an effect of the reduction in the utilization of dental services (in turn caused by the policies of COVID-19 mitigation/suppression), the research presented in this paper will examine the accounting and operations of a dental practice in order to initially assess the reduction in dental service volume and the increases in the dental treatment costs, and therefore the impact on economic performance. This approach is crucial in those countries, such as Italy, in which the provision of dental services is almost totally managed in the private sector. Indeed, according to a classification of the healthcare provision system relating to European countries, provided by Widstrom and Eaton (2004), the Italian system has adopted a “Southern European Model” where the private sector plays a leading role in the provision of dental services, whereas in Germany a Bismarkian insurance system prevails. The private sector in Italy accounts for more than 89% of oral healthcare, as highlighted in a 2016 report compiled by the Italian Government: on average a mere 10% of healthcare out-of-pocket costs are covered by health insurance [7,8]. Whilst the scenario under consideration is different, the findings outlined in this paper are in agreement with those of Schwendicke et al. [6] in that they confirm a stronger impact on economic performance (also taking into account the increase in costs). Guidelines relating to mitigating this impact will also be suggested.

The authors of this paper contend that its innovation lies in its multidisciplinary approach combining knowledge from the field of operations management (McLaughlin et al. [9]), accounting (Warren et al. [10]), revenue management (Birkenshaw Garabelli [11]) and dental practice management.

In conclusion, it is hoped that the research findings will assist the dentist-entrepreneur to become aware of the consequences of their decisions on the economic and operative sides, thereby providing revealing insights for managing post-COVID professional activities. This is in addition to alerting the policy maker as to the threats to public health, which could derive from short-sighted economic measures.

## 2. Materials and Methods

The starting point of the study was an analysis of the most common dental treatments (Table 1).

These require an average treatment time, as documented in the literature, and as displayed in the list of the dental charges of the larger Italian associations in the field [12]. Table 1 describes the risk level, which is associated with each treatment. This depends on the exposure to droplets produced during the procedure. As proposed in a recent article [13], each level of risk is attributed to the single procedure based on: (1) contact with saliva, (2) contact with blood, (3) the use of instruments producing low levels of spray/aerosol (air-water syringes), (4) the production of high levels of spray/aerosol produced by rotating, ultrasound and piezoelectric tools, and (5) the duration of the procedure (operator-dependent). This analysis was necessary for classifying the various treatments according to criteria selected in function of the analysis to be undertaken. The revised recommendations regarding the prevention of COVID-19 for dental activities fundamentally produced two economic effects:

- an increase in the time period between subsequent treatments (time Δ*t*), reducing the time available in a given time period (generally one year) for the treatment, and
- an increase in fixed costs (e.g., the adapting of air-conditioning plants) and variable costs (e.g., personal protective equipment (PPE).

The increase in the time period between subsequent treatments is a general measure, which has been introduced to sufficiently air the dentist’s room. As a further consequence, this may cause an increase in the incidence of fixed costs per unit of treatment (fixed costs have to be allocated to each production output; that is, each treatment, to compute the cost per unit) due to the reduction in the maximum business volume. 

**Figure 1:**
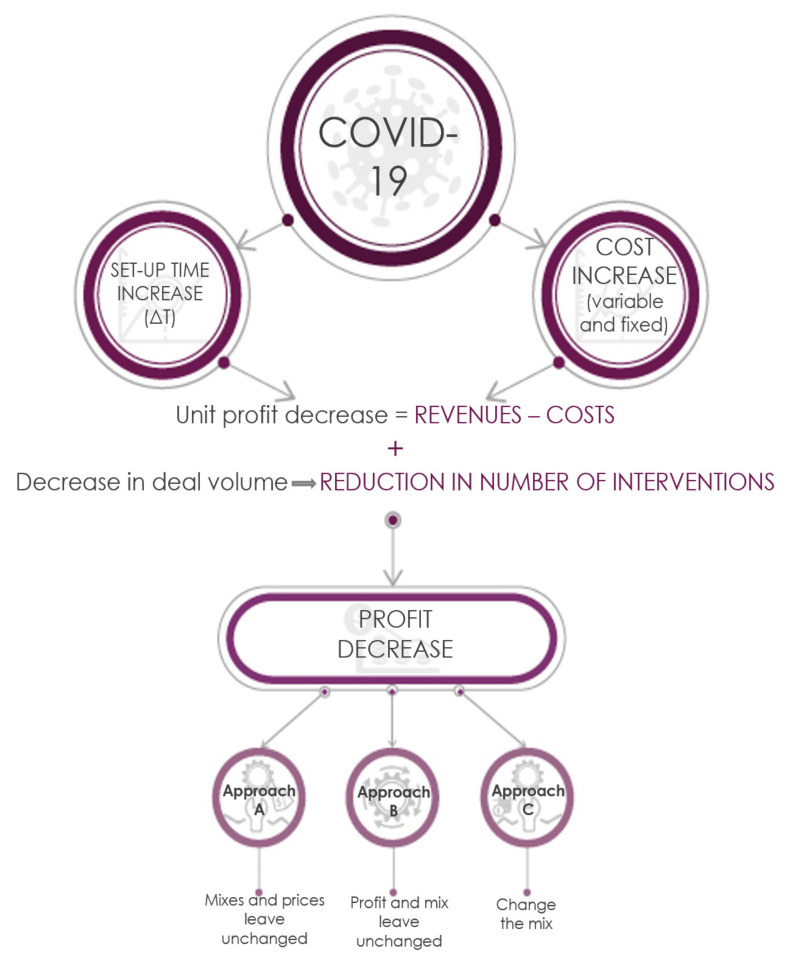
The effect of anti-COVID recommendations on the markup of a dental surgery and three possible approaches for making economic choices.

These effects can cause a reduction in total markup because per unit markup (the difference between earnings and the costs of a single treatment) and the total business volume (in terms of number of treatments) are reduced. Figure 1 summarises the effects of COVID-19 on the dentist’s markup and three possible approaches to the economic choices of the owner of a dental practice.

### 2.1. Methodology

The following are discussed in Appendix A: the variables used in the following sections and the relationship between the total treatment volume before (*V*) and after (V*), the anti-COVID measures adopted, between treatment costs before (*c*) and after (c*), the adoption of anti-COVID measures. In general, the superscript “*” denotes the considered variable after the anti-COVID measures. Moreover, it provides an insight into the pricing of treatments and the effects of the anti-COVID measures, which have been adopted. These effects will be more marked for treatment with reduced pre-COVID working times and higher associated risks. 

In order to analytically assess the economic impact of the post-COVID changes, of paramount importance is an estimate of the loss in markup, which would be incurred in leaving the dental charges for treatment unchanged (Approach A). Thereafter, the trend in the economic performance of the dental surgery was studied by varying the two operating decision variables available to the dentist: pricing (Approach B); and the treatment volumes available within the time limits and resource constraints (Approach C, developed in Appendix B). Following Approach B, the trend in variation in dental charges applicable in the post-COVID era was analysed by keeping the pre-COVID markup constant. Following Approach C, the choice of the treatment mix of the different treatments available, leading to different economic results (markup and turnover), was analysed. In brief, an assessment of the economic impact of the recommendations for containing COVID-19 was performed according to three possible approaches, as described below:

A. Leave the dental charges of single treatments unchanged with a resulting loss in markup;
B. Increase the charge of single treatments in order to maintain unvaried markup; and
C. Modify the dental treatment mix available (*product mix*) so as to contain the loss in economic performance.

### 2.2. Operating Phases

**Figure 2:**
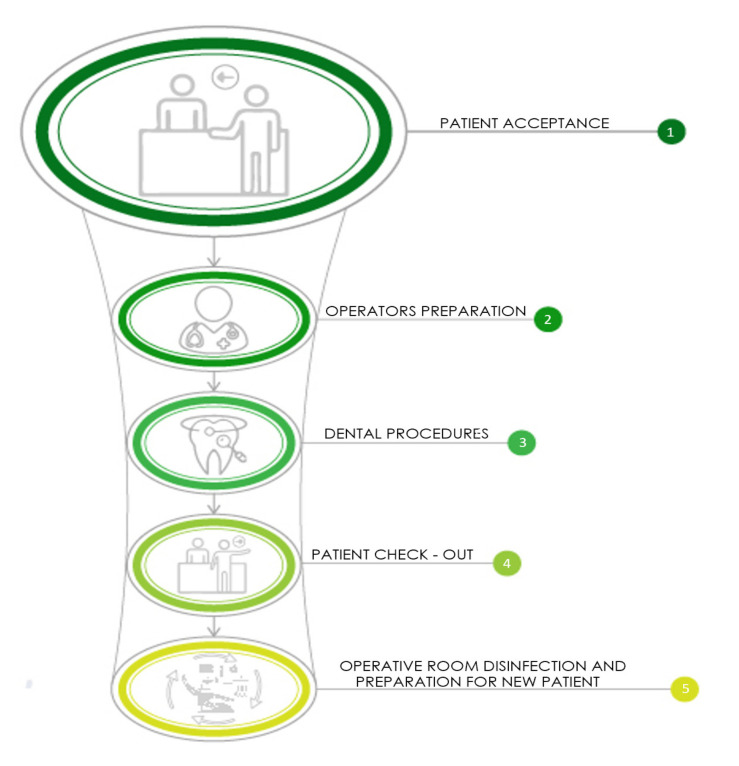
Five operating phases: phase 1, receiving the patient; phase 2, preparing the dental staff and patient; phase 3, treatment, phase 4, patient check out; and phase 5, preparing the room for the next patient.

In order to obtain an initial production performance analysis prior to an economic performance analysis of a given dental surgery, both considered necessary for assessing the COVID impact, it can be hypothesized that the patient passes summarily through three stages: reception, treatment and checking out. In effect, there are five operating phases involving the dental team: (1) reception; (2) preparing the dental staff and patient; (3) treatment; (4) check out; and (5) preparing the room for the next patient (Figure 2). 

Phases 2, 3 and 5 deploy the same resources (the dentist’s room and chair; that is, the treatment room (TR)), whilst phases 1 and 4 generally regard the waiting room/secretary’s area. An intuitive conclusion can be reached; that is, that the space deploying the greatest amount of resources is the TR, and it thereby becomes the critical resource or the bottleneck of the dental procedure. It is precisely this bottleneck which determines the production pace; that is, the speed by which the treatments can be performed and, therefore, the treatment volumes. In order to determine the latter, the dentist’s surgery can be here considered as a single-dentist practice or in possession of one TR with the hypothetical maximum use of the TR resource or system saturation.

The various phases involving the TR have a total duration (*t_w_*), which is given by the sum of time required to prepare the dental staff, the effective treatment time (*t_b_*), and the time necessary to make the TR operational between two successive appointments (the set-up time or *t_s_*). Such a time period (*t_s_*) in the post-COVID era has increased by approximately 30 min (15 min for obligatory airing of the TR and an extra 15 min for sanitizing procedures) [1,14]. This incremental time will henceforth be indicated by Δt. The duration of the bottleneck phase will determine the productivity (the number of treatments in any given time period T) of the entire process; in this context, it coincides with the cycle time t_c_ of the process; that is, the time period between two successive treatments (*t_c_* = *t_w_*).

## 3. Results

Analysing the two main Approaches (A and B) produces the following results: 

### 3.1. Approach A (Dental Charges and Product Mix Unchanged)

It can be pondered as to how much the markup loss, the difference between the post-COVID and pre-COVID markup, ($P^{*}-P$) per treatment unit ($V^{*})$ is worth if dental charges remain unchanged (pre-COVID charge, *p* is equal to post-COVID charge, p*):

$$\frac{P^{*}-P}{V^{*}}=\frac{\left(p^{*}-c^{*}\right)\cdot V^{*}-\left(p-c\right)\cdot V}{V^{*}}$$

denoting c* in function of *c* ($c^{*}=z\cdot c$) and V* in function of *V* (V* = kV) as explained in Appendix A, and keeping dental charges unvaried (p* = *p*), the following will be obtained:

$$\frac{P^{*}-P}{V^{*}}=\frac{\left(p-z\cdot c\right)\cdot k\cdot V-\left(p-c\right)\cdot V}{k\cdot V}=\frac{\left(p-z\cdot c\right)\cdot k-\left(p-c\right)}{k}$$

In order to quantify the markup loss in a dimensionless measure, the percentage value as compared to the unit markup $\left(p-c\right)$, which was obtain pre-COVID, can be calculated:

P*−PV*%=P*−PV*p−c=p−z·c·k−p−ck·p−c=p−z·c·kk·p−c−1k

**Figure 3:**
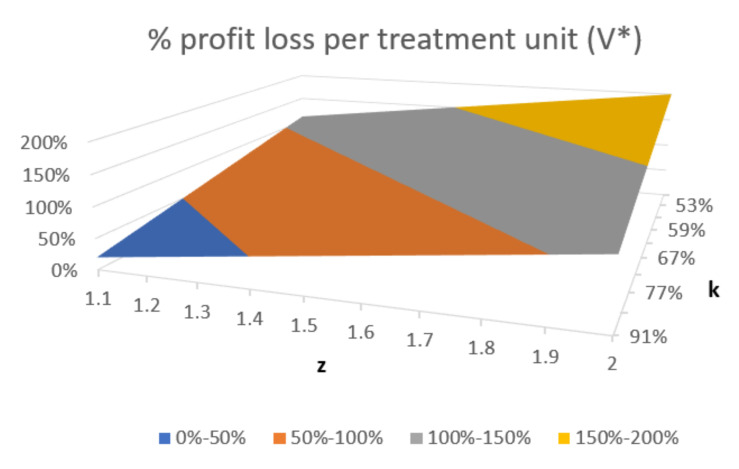
Percentage markup loss per treatment unit (V*) of an increase in costs (*z*) and a reduction in treatment volumes (*k*), keeping dental charges unchanged (*p* = 200 monetary units; *c* = 100 monetary units).

Figure 3 illustrates the trend in percentage loss of markup per treatment unit when costs are increased (*z*) with a decrease in treatment volume (*k*), keeping the dental charges unchanged. As input data, the following can be assumed: an average dental charge, *p*, equal to 200 monetary units and a unit cost C equal to 100 monetary units. The choice of expressing the relationship between operating pre- and post-COVID variables, which have changed due to the new recommendations by means of dimensionless parameters, renders the trend in Figure 3 independent of the specific values of the assumed inputs.

As can be noted in Figure 3, marked reductions in volumes (*k* = 50%) and substantial increases in costs (*z* = 2) will lead to a markup loss of 200%; markup losses (the difference between post and pre-COVID profit) greater than 100% (represented by the grey and yellow areas in Figure 3) imply a loss (where costs exceed earnings). 

### 3.2. Approach B (Markup and Product Mix Unchanged)

The dentist can manage the change in variable costs and working time (and, therefore, the same amount of resources used, i.e., their productive capacity) by intervening in pricing. For example, it could be decided to vary the average dental charges in order to leave the markup unvaried. By how much should the average post-COVID (p*) dental charge increase, compared to the pre-COVID dental charges (*p*) if we wish to leave the markup unchanged? In the latter case (markup unchanged), the dental charges p* must be sufficient to guarantee the following parity of pre- and post-COVID markup:

$$\left(p^{*}-c^{*}\right)\cdot V^{*}=\left(p-c\right)\cdot V$$

from which it follows that the dental charges p* must be:

$$p^{*}=\frac{\left(p-c\right)\cdot V+c^{*}\cdot V^{*}}{V^{*}}=\frac{\left(p-c\right)}{k}+c^{*}$$

and, therefore, the percentage variation compared with the pre-COVID dental charges will be:

p*−pp=1k−1+z·c−1k·cp=1k−1+c·z−1kp

**Figure 4:**
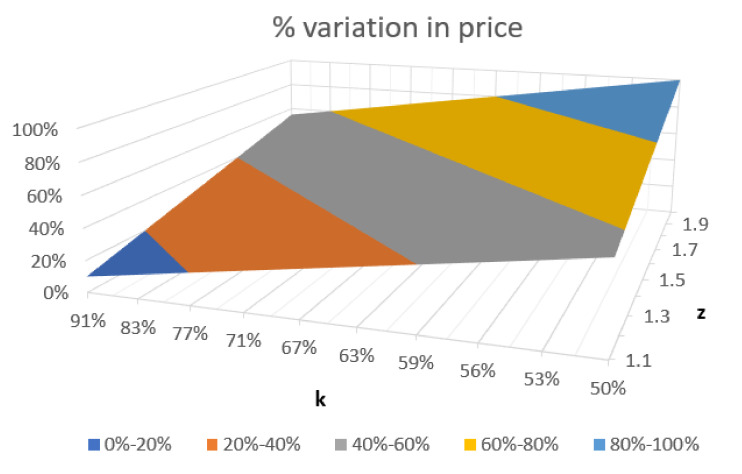
Percentage variation in dental charges of varying an increase in costs (*z*) and a reduction in the number of treatments (*k*), keeping the markup unchanged (*p* = 200 monetary units; *c* = 100 monetary units).

Figure 4 shows the percentage variation in dental charges when modifying an increase in costs (*z*) and a reduction in the number of treatments (*k*), by keeping the markup unvaried. As input data, an average dental charge *p*, equal to 200 monetary units, and a unit cost, equal to 100 monetary units, is also assumed here. As with Figure 4, the choice of expressing the relationship between operating pre- and post-COVID variables, which change due to the new recommendations, in terms of dimensionless parameters, renders the trend in Figure 4 independent of the specific values of the assumed inputs.

It can be noted in Figure 4 that marked reductions in treatment volumes and substantial increase in costs can lead to a doubling of dental charges (percentage variation in dental charges = 100%). Thus far, the dentist-entrepreneur has left the treatment mix with Approaches A and B unchanged; Approach C (Appendix B) investigates another opportunity, demonstrating how changing the mix by increasing certain treatments might impact on markup and turnover, which this research has indicated as the most profitable. After a comment regarding the managerial implications of Approaches A B, the Discussion section will elaborate on Approach C. 

## 4. Discussion

The anti-COVID recommendations promulgated by governments have dramatically changed the competitive scenario for dental practice owners. In order to survive, they need to react quickly to a changing landscape and be aware of the economic and operational consequences of their decisions. The authors of this paper hold that the model presented in this paper is an easy tool for measuring the economic consequences of these changes. It is also hoped that the results of this research will indicate the way for the dentist-entrepreneur to measure the profitability of given treatments in the post-COVID era, thereby providing support in the decision-making of prices and treatment mix. The analysis performed on Approaches A and B enables the dentist to assess the total markup loss, and, on the basis of resources of slack financial assets (an excess in financial assets), to evaluate their resilience; that is, the capacity to resist change, as imposed in the post-COVID era. According to Reeves et al. [15], the application of the principle of resilience in developing policies is one of the 12 principles with which to guide a business through the coronavirus crisis. An increase in fixed costs is a long-term increase (whose duration is equal to the useful life of new investments) whilst an increase in variable costs could be temporary; however, this situation could change drastically with the eradication of the disease. The dentist must, therefore, assess whether the capacity of the activity can cope with long- and short-term investments, deciding whether to maintain dental charges unchanged and, therefore, assess the expected losses and their sustainability. The alternative is to pass on the costs of the post-COVID effects to the patients, deciding whether to keep their own markup unaltered by increasing dental charges. 

Thus, the dentist has at their disposal a range of dental charges (from those pre-COVID to post-COVID, the latter which guarantees unvaried markup), which facilitates the assessing of the appropriacy of greatly increasing pre-COVID dental charges, according to the financial resources available. This decision necessitates a compromise between the first alternative (whereby the dental surgery absorbs the economic effect of COVID) and the second (whereby the market absorbs these effects).

The loss in markup will be even greater for smaller dental practice owners (like those of single treatment room), who are usually unable to exploit economies of scale (a reduction in average production costs of increasing the productive capacity by, for example, duplicating the number of a dentist’s rooms). 

Further investigations would be directed towards an assessment of the opportunities of, on the one hand, modifying treatment pricing (for example, differentiating the percentage mark up on costs), and, on the other hand, increasing the volumes of product mix of those treatments, which have been affected less by the anti-COVID recommendations. As previously expounded, these treatments have the lowest risk and Δ*t/t_w_*. The latter hypothesis (varying the mix of treatments) has been investigated in Approach C (Appendix B); it suggests that the dentist might reconsider their operating choices in the pre-COVID era.

Let us assume that, in the pre-COVID era, the trade-off between satisfying the market demand for a set of treatment types and the constraints of available resources (human and technological) led to the adoption of a business model with an assigned time frame for each typology of treatment considered (MIX2 in Appendix B). The risk is that myopic profit maximization could lead the dentist-entrepreneur to eliminate certain treatments in order to mitigate the economic impact of the anti-COVID measures, which have been adopted. If, for example, the compromise between satisfying the market demand for the treatment types considered in the Approach C (Table 2) and the constraints of available human and technological resources (analysed from an economic point of view) had led to the adoption of a business model with an assigned time frame for each typology of treatment considered (MIX2 in Appendix B) in the pre-COVID era, an assessment or elimination of certain treatments could be made in the post-COVID era. For example, this business model would refer to dental practices which make use of external specialists (corresponding to specified treatments types) in a specified time period (once or twice per week). The eliminated treatments would no longer be profitable or the dentist could consider changing the business model, approaching that with the same treatment volumes for each typology of treatment (MIX1 in Appendix B), which is based on a greater flexibility of resources.

In addition to the proposed approaches discussed in this paper, it would be possible to adopt a comprehensive empirical solution in concentrating a greater number of treatments regarding the same patient in one sitting with a net reduction in the expenditure and the total time between one patient and the other. Lastly, the benefit of duplicating the so-called bottleneck resources could be assessed, that is, to have at least two TRs functioning independently of each other. However, this latter solution necessitates an audit of a break-even volume; that is, that minimum treatment volume which equalises costs with earnings and, therefore, the level under which markup is negative. Indeed, if the treatment request is less than the break-even volume, the solution would not be economically advantageous. Unfortunately, this could be a probable result from the moment when recent investigations have revealed a drop in the request for dental care [16]. 

On a brighter note, the Boston Consulting Group recently surveyed approximately 7000 patients nationwide. The results of this survey indicated that providers of health care services can influence the conditions affecting a patient’s willingness to reschedule delayed care. Addressing concerns such as “The procedures are clear to me,” and “The location is certified free of COVID-19” are within the control of healthcare providers [17]. A contraction of demand could only exacerbate the analyses of the economic performance regarding Approaches A and B, and, on reflection, Approach C: the three Approaches are based on a full deployment of resources. If such a deployment of resources should diminish, on account of a drop-in demand, the impact of increased fixed costs would probably increase, in addition to a reduction in volumes. This, in turn, would lead to a further loss in markup and turnover or a further increase in dental charges in keeping markup constant. However, this latter solution (an increase in dental charges) must contemplate a net contraction in the economic situation and a reduced willingness of the patient to pay. These demand side issues have not been tackled in this paper, the latter being strictly related to the specific market associated with the public health system in a given context. The change in the willingness to pay will modify the patient’s behaviour regarding the purchasing of dental services; the patient may then be obliged to seek financing (a solution generally offered by franchises) or request delayed payment terms for treatment which cannot be postponed. Such a request would lead to further repercussions on the finances of the dentist practice, which may, therefore, increase the financial requirements of working capital or liquidity, which are necessary to keep the dentist practice operational. Manson, in a Harvard Business Review Insight, has outlined a survival strategy for small businesses in the COVID era: the securing of liquidity, the ensuring of access to capital and the engagement with policy-makers are considered to be the three elements which small businesses need to survive in the coronavirus crisis [18].

Another consideration in this analysis regards the assumption of standalone treatments: indeed, complex treatments require multiple appointments which cannot be eliminated from the mix, which is on offer without eliminating the completion of the treatment. In this case it could be helpful to consider a further mix made of complex treatments, including all the single treatments required. 

Finally, consideration should be made regarding the financial support of the policymaker in adjusting to the revised, protective recommendations. Such an injection of liquidity, also by means of guaranteed loans or non-repayable grants, would permit the moderation of a natural increase in dental charges which, in turn, would boost the choice of seeking medical care where those dental charges are lower (for example, health tourism). A potential risk here would not only be a reduction in demand and, therefore, business volume, but it could also have a feedback effect on the public health system in the country in question. 

## 5. Conclusions

The authors of this paper aspire to provide useful managerial insights which can assist the dentist-entrepreneur to become aware of the boundaries of the economic consequences of governmental measures in containing viral infection.

## Figures and Tables

**Table 1:** Analysis of the most common dental and oral medicine treatments.

Procedure	Timing and Risk-Level Post-COVID-19		
	Duration of Procedure (Minutes) + 45 min after Each Patient	Risk-Level	Charges from the National Italian Price List (in Monetary Units) *
Orthodontic checks	30–60	Low	1 **: Orthodontic interceptive therapy: 1500/year Fixed orthodontic therapy: 2000/year Lingual orthodontics: 3000/yearInvisible aligners (complete therapy): 4500/year
Manual reduction of dislocation of the jaw	≤30	Low	150
Mobile/fixed orthodontic appliance positioning	>60	Low	**1**
X-rays	≤30	Low	Intraoral X-ray: 30 Intraoral X-ray status: 200 Orthopantomography: 80 Lateral Teleradiograph: 80
Periodontal therapy	≤30	Low	100
Topical treatment of dental hypersensitivity and caries prophylaxis	≤30	Low	100
Test of night guards	≤30	Low	800
Impression	≤30	Low	
Dental prosthesis tests, positioning and adaptation (temporary/definitive, removable/fixed)	30–60	Low	**2:** Gold-porcelain/zirconia crown750
Biopsy	≤30	Low	200
Bone graft (autogenous/biocompatible material) without rotating tools	>60	Low	400–700 (with membrane)
Mucogingival surgery (quadrant)	30–60	Low	600
Subgingival curettage without rotating tools (quadrant)	30–60	Low	500
Removal of cysts or small benign neoplasms	30–60	Low	500
Surgical medication	≤30	Low	
Oral minor surgery (e.g., abscess incision, frenulectomy, frenulotomy)	≤30	Low	150–300
Salivary stone removal	≤30	Low	150–500
Extraction without rotating tools	30–60	Low	130
Gingivectomy/gingivoplasty	≤30	Low	300
Endodontic treatment (1 root) with rubber dam (in subsequent appointment after access cavity)	≤30	Low	**3:** 180 + 100 (for each additional canal)
Pulp-capping, pulpotomy, pulpectomy (in subsequent appointment after access cavity) with rubber dam	30–60	Low	130
Bleaching	>60	Medium	450
Orthodontic splinting (1 dental arch)	≤30	Medium	200
Orthodontic splinting (2 dental arches)	30–60	Medium	400
Periodontal splinting (1 dental arch)	≤30	Medium	200
Periodontal splinting (2 dental arch)	30–60	Medium	400
Intra-oral examination	≤30	Medium	100
Tartar removal	30–60	High	110
Extraction with rotating tools	30–60	High	150–300
Sinus lift	>60	High	600–1200
Cavity access (rotating instruments)	≤30	High	**3**
Implantology	>60	High	1200
Subgingival curettage (quadrant) (rotating tools)	≤30	High	500
Resective/regenerative bone surgery (rotating tools)	>60	High	750–900
Rhizectomy/rhizotomy (rotating tools)	30–60	High	150–300
Sealing of dental grooves	≤30	High	50 (for each tooth)
Apicectomy with retrograde filling	>60	High	300–500
Autologous bone harvest (rotating tools)	≤30	High	300–450
Abutment tooth preparation	≤30	High	2
Odontoplasty (1 tooth)	≤30	High	100–250 (for each tooth)
Simple/complex filling using rotating tools	30–60	High	150–250
Extraction of impacted tooth with rotating tools	>60	High	300–500

* The charges reported in the far right column refer to the maximum values of the ANDI (Italian association of Dentists) 2009 price list [12]. ** The numbers 1, 2 and 3 in bold in the far right column indicate that the procedure described is not an independent service (with a single price) but it is always associated with other services with the same code number, where an all-encompassing charge is applied.

**Table 2:** Grouping of the dental treatments on the basis of the time and risk variables, and the risk of contagion.

Dental Treatments		Risk-Level		
		Low	Medium	High
	*t_w_* ≤ 45’(low)	Manual reduction of dislocation of the jaw X-rays Topical treatment of dental hypersensitivity and caries prophylaxis Biopsy Oral minor surgery (e.g., abscess incision, frenulectomy, frenulotomy) Salivary stone removal Gingivectomy/gingivoplasty	Orthodontic splinting (1 dental arch) Periodontal splinting (1 dental arch) Intraoral examination	Subgingival curettage (quadrant) (rotating tools) Sealing of dental grooves Abutment tooth preparation Odontoplasty (1 tooth)
TIMING (*t_w_*)	45’ < *t_w_* ≤ 75’(medium)	Mucogingival surgery (quadrant) Subgingival curettage without rotating tools (quadrant) Removal of cysts or small benign neoplasms Tooth extraction without rotating tools	Orthodontic splinting (2 dental arches) Periodontal splinting (2 dental arches)	Tartar removal Extraction with rotating tools Simple/complex filling using rotating tools
	*t_w_* > 75’(high)	Bone graft (autogenous/biocompatible material) without rotating tools	Bleaching	Sinus lift Implantology Resective/regenerative bone surgery (rotating tools) Apicectomy with retrograde filling Extraction of impacted tooth with rotating tools

**Figure A1:**
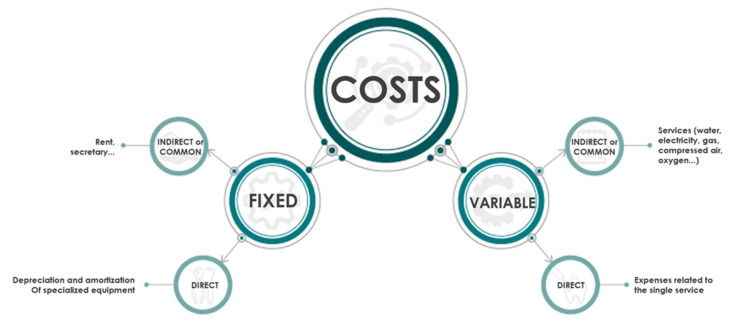
Classification and description of the main costs in a dental surgery.

## Appendix A

### Appendix A.1. Operating Phases

In order to arrive at an initial product performance analysis prior to an economic performance analysis of a dentist’s surgery—both considered necessary for assessing the COVID impact—it can be hypothesized, in a very simple diagram, that the patient passes through three stages: reception, treatment and checking out. In effect, there are five operating phases involving the dental team: phase 1, receiving the patient; phase 2, preparing the dental staff and patient; phase 3, treatment; phase 4, patient check out; and phase 5, preparing the room for the next patient (Figure 2). Phases 2, 3 and 5 deploy the same resources (the dentist’s room and chair—treatment room (TR)), whilst phases 1 and 4 generally regard the waiting room/secretary’s area. An intuitive conclusion can be reached at this juncture: the space deploying the greatest amount of resources is the TR, and it thereby becomes the critical resource or the bottleneck of the dental procedure. And it is precisely this bottleneck which determines the production pace, that is, the speed by which the treatments can be performed and, therefore, the treatment volumes. In order to determine the latter, the dentist’s surgery can be here considered as a single-dentist practice or in possession of one TR with the hypothetical maximum use of the TR resource or system saturation.

The various phases involving the TR have a total duration (*t_w_*), which is given by the sum of time required to prepare the dental staff, the effective treatment time (*t_b_*), and the time necessary to make the TR operational between two successive appointments (the set up time or *t_s_*). Such a time period (*t_s_*) in the post-COVID era has increased by approximately 30 min (15 min for obligatory airing of the OR and an extra 15 min for sanitizing procedures). This incremental time will henceforth be indicated by Δ*t*. The duration of the bottleneck phase will determine the productivity (the number of treatments in any given time period *T*) of the entire process; in this context, it coincides with the cycle time *t_c_* of the process; that is, the time period between two successive treatments (*t_c_*_=_ *t_w_*).

### Appendix A.2. An Increase in Time

The impact of an increase in time (Δ*t*) between two successive treatments will be initially assessed. With *T* representing the extent of the availability of the TR (generally *T* in hours = 220 productive days * 8 h/day = 1760 h) and hypothesizing 100% use, the number of treatments (*V*), which can be performed in a given timeframe, is *T/t_c_.* Indicating the post-COVID variable subscript with a * (having activated anti-COVID protective measures), *t_c_* = t_c_ +* Δ*t*, where Δ*t* is equal to 30 min. This new *t_c_** will determine a different number of treatments, *V** (*V** = *T/t_c_** = *T*/(*t_c_* + Δ*t*)), which is less than V. By analysing the main treatments under consideration, the following can be hypothesized: an average duration of *t_b_* equal to 45 min, a *t_s_* equal to 15 min and, as a consequence, *t_c_* is equal to 60 min.

$$V=\frac{T}{t_{c}}$$

$$V^{*}=\frac{T}{t_{c}^{*}}=\frac{T}{t_{c}+\Delta t}=\frac{T/t_{c}}{t_{c}/t_{c}+\Delta t/t_{c}}=\frac{V}{1+\Delta t/t_{c}}\;=k*V$$

where *k* (lower than 1) indicates the contraction coefficient of the production capacity, which expresses the post-COVID treatment volumes in function of the pre-COVID treatment volumes.

$$k=\frac{1}{1+\Delta t/t_{c}}\;$$

As can be noted, the contraction in treatment volumes, represented by the parameter k (always less than 1), is proportional to the relationship between the increase in total working time and the total pre-COVID time.

Assuming the numerical values introduced above, the post-COVID volume of treatments becomes:

$$V^{*}=\frac{V}{1+30\prime /60\prime }=\frac{2}{3}\cdot V$$

therefore *k* = 2/3. It is evident that k will vary with *t_c_*: for example, if *t_c_* = 45′ and Δt is equal to 30 min, *k* = 3/5.

### Appendix A.3. Markup

Having estimated the impact of the pre- and post-COVID-19 recommendations on the number of treatments, it is now possible to estimate pre- and post-COVID markup: let us indicate markup as *P, c* the average unit costs and *p* the average unit dental charge, where the average cost and dental charges refer to the average weighted cost and dental charges, respectively, and where the weighting is determined by the relative volume of the single treatment type. In general terms, a number of R treatments can be hypothesized and the generic treatment *r* with *r* = 1, 2…. *R* will have a unit cost *c_r_* with a dental charge *p_r_* and a volume *V_r_*. The cost and the average dental charges can be expressed thus:

$$c=\frac{\sum_{r=1}^{R}c_{r}\cdot V_{r}}{\sum_{r=1}^{R}V_{r}}$$

where $\sum_{r=1}^{R}V_{r}=V$ and $mix_{r}=\frac{V_{r}}{V}$ indicates the percentage of the treatment type *r*, which is performed on all the treatments, the same holds for the average dental charges:

$$p=\frac{\sum_{r=1}^{R}p_{r}\cdot V_{r}}{\sum_{r=1}^{R}V_{r}}$$

the total markup *P* will be

$$P=\left(p-c\right)\cdot V$$

In the post-COVID era, the costs of single treatments will increase (due to increases in fixed and variable costs), whilst the volumes, (hypothesized to be equal to the productive capacity of the surgery) will diminish. New costs *c** will be indicated in function of old costs *c*, by means of a multiplication factor *z*:

$$c^{*}=z\cdot c$$

The introduction of the *k* and *z* parameters permits us to conduct a parametric analysis of the economic effects of COVID-19 and to define the trends in economic performance, which disregard the specific numerical values used, thereby generalising the results obtained.

### Appendix A.4. Treatment Pricing

It is necessary at this juncture to make some observations regarding the pricing, the estimate of treatment costs (which determines dental charges) and increases in both items. A pricing method, based on a percentage addition of the costs, can be assumed; that is, the so-called *price-making* (as opposed to price-taking, which fixes the dental charges based on the current market dental charges). Price-making, therefore, requires an estimate of the treatment costs. The challenge of estimating the costs arises because a dental practice offers different treatments. If treatment was of one type, it would be sufficient to total all the costs and divide by the volume of the treatments on offer. Costs can be divided into fixed and variable costs (according to their behaviour in function of the treatment volume achieved: the former are constant within a certain range of volume, the latter increase proportionately) or direct and indirect costs. Indirect costs are subject to the challenge of allocation, that is, quantifying the costs assigned to single treatments, and sharing the resources generating the common cost [19]. Assuming a single treatment as a *cost object* and the number of treatments undertaken throughout the year as *cost-driver*, of relevance to this paper are indirect (or common), fixed and variable costs (i.e., overheads, amortization and depreciation of equipment and buildings), which are allocated to identifying the cost of a single treatment (Figure A1).

A functional driver, related to the number of treatments (volume) for distributing costs in professional settings, is the total treatment time. It can, therefore, be assumed that the cost of common resources is absorbed on the basis of the time using those resources. The post-COVID scenario involves two main observations regarding the impact of fixed costs on a singular treatment:

- Fixed common costs will increase following preventative recommendations (regarding, for example, the air-conditioning plant); and
- The number of treatments will diminish due to an increase in the working time, thereby changing the driver disproportionally. This implies that some treatments will absorb greater and lesser costs in percentage terms.

These observations imply an increase in fixed, common costs, which will not be the same for all treatments: reduced pre-COVID working times will be more penalised by the increase in the set-up time, which remains constant (30 min) for all treatments.

Referring to variable costs, the post-COVID impact involves the PPE used; its total cost will vary on the basis of the risk level associated with a particular treatment [20]. Table A1 shows the variables and their description, hitherto introduced, and which will be used in this study. It should be noted that the variables referring to times and k and z parameters can, if necessary, also become subscripts, thereby specifying the treatment to which they refer.

**Table A1:** Variables used in the analysis. The symbol $\sum_{r}x_{r}$ indicates the sum of generic variables $x_{r}$, attributing all possible values to *r* (*r* varies from 1 to *R* in this study).

Variables Used in the Analysis	Description
*CF*	pre-COVID fixed cost in period T
Δ*CF*	increase in fixed costs
*CF* = CF +* Δ*CF*	post-COVID fixed cost in period T
*R*	number of treatments
*cf_r_*	pre-COVID unit fixed cost of *r*-th treatment
*cv_r_*	pre-COVID variable unit cost of *r*-th treatment
*c_r_ = cf_r_+ cv_r_*	pre-COVID unit cost of *r*-th treatment
Δ*cf_r_*	increase in fixed unit costs of *r*-th treatment
*cf_r_ * = cf_r_+* Δ*cf_r_*	post-COVID fixed unit cost of *r*-th treatment
Δ*cv_r_*	increase in variable unit costs of *r*-th treatment
*cv_r_ * = cv_r_ +* Δ*cv_r_*	post-COVID variable unit costs of *r*-th treatment
*c_r_* = cf_r_*+ cV_r_**	post-COVID unit cost of *r*-th treatment
*p_r_*	pre-COVID dental charge of *r*-th treatment
*p_r_**	post-COVID dental charge of *r*-th treatment
*V_r_*	number of pre-COVID *r*-th treatment
*V_r_**	number of post-COVID *r*-th treatment
$V=\sum V_{r}$	total volume of pre-COVID treatments
$V^{*}=\sum V_{r}{}^{*}$	total volume of post-COVID treatments
$P=\sum V_{r}\cdot p_{r}$	total pre-COVID markup from the sum of markup for each treatment
$P^{*}=\sum V_{r}{}^{*}\cdot p_{r}{}^{*}$	total post-COVID markup from the sum of markup for each treatment
T	Timeframe for analysis: 1 year
$mix_{r}=\frac{V_{r}}{\sum V_{r}}$	percentage volume of *r*-th treatment on total volume
$mix_{r}^{*}=\frac{V_{r}^{*}}{V^{*}}$	percentage volume of *r*-th treatment on total post-COVID volume
*z*	increase in post-COVID unit costs
*K*	reduction in post-COVID volumes
*t_b_*	preparation time for staff and treatment slot
*t_s_*	time necessary to render the OR operational between successive treatments
*t_w_*	total treatment duration
*t_c_*	cycle time or time interval between completing two successive treatments
Δ*t*	increase in time *t_s_*, resulting from anti-COVID recommendations

## Appendix B

*Approach C* (analysis and application of product mixes).

Hitherto, reference has been made to a total assessment of the productive-economic performance in dental practices. Consideration has also been made of a typical treatment, also those with equivalent treatment times for a typical treatment. However, the results shown in Figure 3 and Figure 4 will vary in function of the characteristics of the treatment under consideration. A meticulous analysis of a single treatment would probably prove to be ineffective; thus, it is considered preferable to analyse homogeneous groups of treatment. It is evident that this criterion of homogeneity must be in function of the analysis under consideration.

On the basis of the considerations regarding the economic effects of the anti-COVID recommendations concerning dental procedures, there are two criteria for grouping single treatments:

- On the one hand, the working time (which impacts on the volumes achievable, as previously demonstrated; refer to the *Increase in time* sub-section above), and the fixed unit cost (to be elaborated below)
- And, on the other, the risk level of the treatment (which impacts on the variable costs).

Nine treatment groups (3 × 3) will be obtained (Table 2), subject to the following: in the interests of simplicity, without compromising the quality and generalisability of the results, considering the three levels for the working time criterion (namely *t_w_*_1_, *t_w_*_2_, *t_w_*_3_) and the three levels regarding risk ([13]; namely l, m, h: low, medium and high respectively). Referring to Table 2, treatments with *t_w_* ≤ 45’ belong to the average-working-time-group, which is equal to *t_w_*_1_; treatments with 45′ < *t_w_* ≤ 75′ belong to the average-working-time-equal-to-t_w2_ group _,_ and treatments with *t_w_* > 75′ belong to the average-working-time-equal-to-t_w3-_group. The treatments considered are stand alone, i.e., they are provided as single treatments and they are, therefore, independent each other.

The percentage value of the treatment volume of a certain type, compared with the total volume, will be indicated by the following: mix*_ij_* con *i* = *t_w_*_1_, *t_w_*_2_, *t_w_*_3_ e *j* = l, m, h. Table A2 shows 9 possible mixes, derived from the choices made by bearing in mind the levels of working time and risk level.

**Table A2:** The mix*_ij_* under consideration yields 9 treatment types by classifying the treatments on the basis of the working time (*i*) and the risk level (*j*) (as in Table 2).

	*j* = l	*j* = m	*j* = h
*i* = *t_w_*_1_	mix*_tw_*_1l_	mix*_tw_*_1m_	mix*_tw_*_1h_
*i* = *t_w_*_2_	mix*_tw_*_2l_	mix*_tw_*_2m_	mix*_tw_*_2h_
*i* = *t_w_*_3_	mix*_tw_*_3l_	mix*_tw_*_3m_	mix*_tw_*_3h_

Table A3 describes a possible distribution of percentage volumes in the 9 highlighted treatment groups: in this example, $\mathrm{mix}{\;}_{t_{w1}m}$ = 15% means that the treatment volumes, with a working time equal to *t_w_*_1_ and an average risk, (*j* = m), comprise 15% of total treatment volumes.

**Table A3:** A numeric example of Table A2.

mix*_ij_*	*j* = l	*j* = m	*j* = h
*i* = *t_w_*_1_	8%	15%	12%
*i* = *t_w_*_2_	10%	11%	11%
*i* = *t_w_*_3_	9%	12%	12%

With the necessity of transitioning to a double index to indicate a treatment group, it is useful to identify a match between the single and double subscripts. Thus, the variables introduced in Table A1 can be used, thereby ensuring a change in the subscript, as proposed in Table A4; the choice is not unambiguous but irrelevant to that which follows.

**Table A4:** The match between a coding mix with a subscript (*r*) and with two subscripts (*ij*).

subscript *r*	1	2	3	4	5	6	7	8	9
subscript *ij*	*t_w_*_1l_	*t_w_*_1m_	*t_w_*_1h_	*t_w_*_2l_	*t_w_*_2m_	*t_w_*_2h_	*t_w_*_3l_	*t_w_*_3m_	*t_w_*_3h_

On the basis of considerations hitherto made, mixes with a greater percentage increase in fixed costs will be the $\mathrm{mix}{\;}_{t_{w1}j}\;$, which deliver a reduced $\Delta t/t_{c_{ij}}$ relationship and, therefore, reduced k; the mixes with a greater percentage increase in variable costs will be the mix*_il_* which, presenting a low risk, require an increase in low or zero variable costs. In order to quantify the post-COVID economic impact relating to the various treatment groups, it is opportune to proceed with numerical examples (given the abundance of variables which do not permit the obtaining of only one solution to the problem) and to make hypotheses regarding the mix under consideration. Specifically, we can assume average values for pre-COVID preparation time and effective treatment time, which are equal to

$$t_{b1}=20\;\min,\;t_{b2}=40\;\min,\;\;t_{b3}=60\;\min$$

Assuming *t_s_* equals to 15 min, the working times will be equal to:

$$t_{w1}=35\;\min,\;t_{w2}=55\;\min,\;\;t_{w3}=75\;\min$$

In the post-COVID era, these times have increased by 30 min to:

$$t_{w1}^{*}=65\;\min,\;t_{w2}^{*}=85\;\min,\;\;t_{w3}^{*}=105\;\min$$

Two mix sets can be hypothesized: the first, indicated as MIX1, will refer to a range of uniform volumes regarding the nine possible treatment groups, therefore, the mix*_il_* = *V*/9/*V* = mix*_il_** = *V**/9/*V** = 11% for treatment type. The average cycle time *t_c_* can be obtained as a weighted average of the three working times, thereby, obtaining a result of 55 min (with the volumes being uniformly distributed). Thus, the total pre-COVID volume can be obtained by dividing the available time T (105,600 min per year) by *t_c_*, thereby_,_ obtaining *V* = 1920 treatments per year. Their single volumes will equal approximately *V*/9 = 213 (completed treatments are rounded up). In the post-COVID era, $t_{c}^{*}$ is 85 min, therefore, *V** = 1242 units (105,600/85) and single volumes will equal 138 units (1242/9).

The second mix, termed MIX2, is based on the hypothesis that a predetermined sum of available time T will be dedicated to each of the 3 typologies, based on the working time (*t*_w1_, *t*_w2_, *t*_w3_). For reasons of simplicity, we can hypothesize that T is uniformly distributed between the 3 groups (therefore, each treatment group *i* (*i* = *t_w_*_1_, *t_w_*_2_, *t_w_*_3_) will have available a time period of T/3 = 105,600 min/3 = 35,200 min). We can further hypothesize a uniform distribution of the risk level pertaining to each of the 3 groups. It is opportune to treat separately the 3 treatment groups (in the MIX2 case), with each having a cycle time equal to the working time. It follows that the pre- and post-COVID volumes for MIX2 will be the following (Table A5 and Table A6).

**Table A5:** Pre-COVID volumes for the 9 treatment groups in MIX2.

*V***_ij_*	*j* = l	*j* = m	*j* = h
*i* = t_w1_	335	213	156
*i* = t_w2_	335	213	156
*i* = t_w3_	335	213	156

**Table A6:** Post-COVID volumes for the 9 treatment groups in MIX2.

*V***_ij_*	*j* = l	*j* = m	*j* = h
*i* = t_w1_	181	138	112
*i* = t_w2_	181	138	112
*i* = t_w3_	181	138	112

The following values relating to the variables described in Table 2 can be considered in the calculation, being of relevance to Approach C. The available time T is always equal to 105,600 min per year and a fixed annual cost (CF), equal to 88,000 monetary units, can be hypothesized (producing an hourly fixed cost of 50 monetary units). Bearing in mind that the average treatment duration is 35, 55 and 75 min for *t_w_*_1*j*_, *t_w_*_2*j*_, *t_w_*_3*j*_ respectively, unit fixed costs equal to approximately 29, 46 and 62 monetary units will be obtained for *cf_w_*_1*j*_, *cf_w_*_2*j*_, *cf_w_*_3*j*_ respectively. Hypothesizing an impact of 35% for *cf_ij_* and 65% for *cv_ij_* on the unit cost (c_ij_), the variable and unit costs (the sum of *cf_ij_* and *cv_ij_*) will be obtained, as shown in Table A7.

**Table A7:** Total variable and unit costs relating to per-COVID treatments.

	*i = t* *_w_*_1_	*i = t* *_w_*_2_	*i = t* *_w_*_1_
*cv_ij_* (monetary units)	54	85	116
*c_ij_* = *cf_ij_* + *cv_ij_* (monetary units)	83	131	179

Hypothesizing a markup of 50%, the pre-COVID dental charges will have the values shown in Table A8. Below it will be seen: (i) that this hypothesis does not invalidate the following results; and (ii) how the variation in markup impacts the economic performance being investigated.

**Table A8:** Pre-COVID treatment dental charges.

	*i = t* *_w_*_1_	*i = t* *_w_*_2_	*i = t* *_w_*_3_
*p_ij_* (monetary units)	125	196	268

A hypothesis can be made regarding an incremental variation in fixed costs in the post-COVID era, necessary to adapt to the COVID-19 preventative regulations (Δ*CF*), equal to 20,000 monetary units. Variable incremental costs (Δ*cv_ij_*) can be assumed equal to 0 for low risk treatments, 10 monetary units relate to medium level risk treatments, and 20 monetary units regard high risk treatments; Δt (an increase in the set up time relating to post-COVID performance) equals 30 min. Consequently, the unit costs will vary, as shown in Table A9, maintaining a markup of 50%, the post-COVID dental charges will obtain the values, as shown in Table A10.

**Table A9:** Post-COVID unit costs of the 9 mixes under consideration.

*c_ij_**	*j* = l	*j* = m	*j* = h
*i = t* *_w_*_1_	121	172	223
*i = t* *_w_*_2_	131	182	233
*i = t* *_w_*_3_	141	192	243

**Table A10:** Post-COVID dental charges for the nine mix typologies under consideration, the average dental charge for the three treatment typologies (l, m, h), based in working times (*p_i_** average) and their percentage variation, compared to the pre-COVID dental charges (Δ*p_i_** average %).

*p_ij_**	*j* = l	*j* = m	*j* = h
*i = t* *_w_*_1_	181	258	335
*i = t* *_w_*_2_	196	273	350
*i = t* *_w_*_2_	211	288	365
*p_i_** average	196	273	350
Δ*p_i_** average %	57%	39%	31%

The values relating to the costs and dental charges in Table A7, Table A8, Table A9 and Table A10 are common to the two mixes (MIX1 and MIX2) under consideration; the effects on economic performance are, however, distinct for the two aforementioned mixes, as shown in Table A11, Table A12, Table A13 and Table A14.

**Table A11:** Percentage of markup loss/treatment unit with dental charges constant for the 9 mixes under consideration in the two mix scenarios (MIX1 and MIX2).

		MIX1				MIX2	
mix*_il_*	144%	117%	105%	mix*_il_*	175%	117%	90%
mix*_im_*	168%	133%	116%	mix*_im_*	199%	133%	101%
mix*_ih_*	192%	148%	127%	mix*_ih_*	223%	148%	113%
average for *i*	168%	133%	116%	average for *i*	199%	133%	101%

**Table A12:** Loss in turnover in monetary units with dental charges constant for the 9 mixes under consideration in the two mix scenarios (MIX1 and MIX2). The total value appears in red in Table A12.

		MIX1					MIX2		
mix*_il_*	9412	14,790	20,168	44,370	mix*_il_*	19,341	14,790	11,973	46,103
mix*_im_*	9412	14,790	20,168	44,370	mix*_im_*	19,341	14,790	11,973	46,103
mix*_ih_*	9412	14,790	20,168	44,370	mix*_ih_*	19,341	14,790	11,973	46,103
total by column	28,235	44,370	60,504	133,109	total by column	58,022	44,370	35,918	138,310
average for *i*	7%	12%	16%	35%	average for *i*	15%	12%	10%	37%

**Table A13:** Revised dental charges in monetary units to maintain markup (of the mixes and therefore totalled) constant for the 9 mixes under consideration in the two mix scenarios (MIX1 and MIX2).

		MIX1				MIX2	
mix*_il_*	185	273	361	mix*_il_*	198	273	348
mix*_im_*	195	283	371	mix*_im_*	208	283	358
mix*_ih_*	205	293	381	mix*_ih_*	218	293	368
average for *i*	195	283	371	average for *i*	208	283	358

**Table A14:** Percentage increase in the dental charges necessary to maintain markup (of the mixes and therefore totaled) constant for the 9 mixes under consideration in the two mix scenarios (MIX1 and MIX2).

		MIX1				MIX2	
	mix_1*j*_	mix_2*j*_	mix_3*j*_		mix_1*j*_	mix_2*j*_	mix_3*j*_
mix*_il_*	48%	39%	35%	mix*_il_*	58%	39%	30%
mix*_im_*	56%	44%	39%	mix*_im_*	66%	44%	34%
mix*_ih_*	64%	49%	42%	mix*_ih_*	74%	49%	38%
average for *i*	56%	44%	39%	average for *i*	66%	44%	34%

It must be remembered that the two mixes, MIX1 and MIX2, refer to the operating choices in terms of volumes of different treatments available. Specifically, MIX1 hypothesizes that the volumes for single treatments may be the same; MIX2 hypothesizes that the time available in the dental surgery for single treatments may also be the same. This means that the volumes of treatments in the MIX2 case may differ since they have different working times for a given treatment. The choice between the two mixes will be based on treatment demand (in turn, market-driven), which will reflect on the organisation of the dental surgery and be compatible with the specialisations demanded by the various treatments.

As can be noted, MIX2 is slightly more penalised in terms of a loss in turnover for the single-dentist practice activity of MIX1 hitherto considered. MIX2 has a k value (a contraction in the number of treatments) varying from 54% to 71%, compared to 65% for MIX1, with z remaining unchanged for the two mixes. This regards the variability (variation range) of MIX2 in terms of economic performance, which is much more marked for MIX1. For example, a percentage increase in the dental changes for MIX1 varies from 35% to 64%, and for MIX2 from 30% to 74%. Thus, the percentage loss in markup for MIX1 is between 105% and 192% whilst that for MIX2 ranges from 90% to 223%. However, the loss in turnover exhibits a variability which is slightly higher for MIX1 than MIX2, being generally higher in absolute terms for MIX2.

If the choice of mixes were to be freely selected, the choice would be to concentrate on the treatments with a lesser k and z; that is, with a longer treatment time and, therefore, a lower Δ*t*/*t_w_* relationship and lower risk (*i* = 3 and *j* = l). In the latter case, the percentage markup loss/treatment unit would be reduced by 90% (keeping the dental charges constant), compared to a range of 101–199%, as recorded in the two numerical examples MIX1 and MIX2. The loss of turnover in monetary units with constant dental charges would diminish to 107,755.10, compared to a loss of the approximate 138,000 monetary units of MIX1, and the approximate 133,000 monetary units of MIX1. In order to keep markup constant, the revised dental charges in monetary units would decrease to 348 monetary units, thereby representing an increase of 30% when compared to the pre-COVID dental charges, with respect to increases varying from 30% to 58% in the two numerical examples MIX1 and MIX2. Intermediate results can be obtained only if the following is considered: those treatments with a lower Δ*t*/*t_wi_* relationship and all three risk levels, or a low risk level and all three working times. Finally, the variation in markup impacts only on the percentage loss in markup/treatment unit with constant dental charges, which increases concomitantly with the reduction in markup.
